# Association Between Activities of Daily Living and Mortality Among Institutionalized Elderly Adults in Japan

**DOI:** 10.2188/jea.JE20110153

**Published:** 2012-11-05

**Authors:** Akemi Nakazawa, Kazutoshi Nakamura, Kaori Kitamura, Yoshiaki Yoshizawa

**Affiliations:** 1 Department of Nursing, Tohto College of Health Sciences, Fukaya, Saitama, Japan; 1 東都医療大学ヒューマンケア学部看護学科; 2 Division of Preventive Medicine, Niigata University Graduate School of Medical and Dental Sciences, Niigata, Japan; 2 新潟大学大学院医歯学総合研究科環境予防医学分野; 3 Niigata Council of Institutions for the Elderly, Niigata, Japan; 3 新潟県老人福祉施設協議会

**Keywords:** activities of daily living, frail elderly, nursing homes, mortality

## Abstract

**Background:**

We assessed the association between activities of daily living (ADL) and mortality among nursing home residents in Japan.

**Methods:**

This 1-year prospective cohort study investigated 8902 elderly adults in 140 nursing homes. Baseline measurements included age, sex, height, weight, body mass index (BMI), ADL, and dementia level. ADL levels were obtained by caregivers, using the Barthel Index (BI), after which total BI scores were calculated (higher scores indicate less dependence). Information on dates of discharge and mortality was also obtained to calculate person-years. The Cox proportional hazards model was used to estimate hazard ratios (HRs).

**Results:**

Mean age was 84.3 years, and mean total BI score was 38.5. The HRs of mortality adjusted for sex, age, BMI, and type of nursing home were 7.6 (95% CI: 3.3–17.8) for those with a BI score of 0 (totally dependent), 3.9 (1.7–9.0) for those with a score of 1 to 10, 3.5 (1.4–8.7) for those with a score of 11 to 40, 2.7 (1.4–5.1) for those with a score of 41 to 70, and 1.3 (0.7–2.4) for those with a score of 71 to 99 (*P* for trend <0.001), as compared with those with a score of 100. Multivariate analysis revealed that BI, sex, age, and BMI were significantly associated with mortality rate.

**Conclusions:**

There was a clear inverse association between ADL level and mortality. In conjunction with other risk factors, ADL level might effectively predict short-term mortality in institutionalized elderly adults.

## Abstract (ja)

**【背景】:**

本研究は、日本の高齢者施設に入居している高齢者のADLと生命予後との関連を明らかにすることを目的とした。

**【方法】:**

新潟県老人福祉施設協議会に所属する高齢者施設のうち研究の同意の得られた140の高齢者施設で生活する8902名の高齢者を対象とした1年間のコホート研究を実施した。ベースラインにおいて年齢、性、身長、体重、BMI、ADLと認知症レベルを調査した。ADLの程度の判定にはBarthel Index(BI)を用い、施設の看護師または介護士の判定によりBIの総得点（高得点ほど自立している）を算出した。退所および死亡した日付より人-年を算出した。Cox比例ハザードモデルを用いて生命予後のハザード比とその95%信頼区間を算出した。

**【結果】:**

対象者の平均年齢は84.3歳、BI総得点の平均は38.5点であった。性、年齢、BMI、施設の種類で調整した生命予後のハザード比は、BI総得点100点（自立）のグループを基準とすると、BI総得点0点（全介助）のグループでは7.6（95%信頼区間：3.3-17.8）、BI総得点1～10点のグループでは3.9（95%信頼区間：1.7-9.0）、BI総得点11～40点のグループでは3.5（95%信頼区間：1.4-8.7）、BI総得点41～70点のグループでは2.7（95%信頼区間：1.4-5.1）、BI総得点71～99点のグループ（有意水準p<0.001）では1.3（95%信頼区間：0.7-2.4）であった。多変量分析では、BI、性、年齢、BMIが生命予後に関連することが明らかになった。

**【結論】:**

ADLレベルと生命予後には明確な負の関連が見られた。他のリスク要因も考慮することにより、ADLレベルは効果的に施設入居高齢者の短期間の生命予後を予測し得る。

## INTRODUCTION

The proportion of people aged 65 years or older in Japan was 23.1% in 2010 and is projected to be as high as 26.9% in 2015.^1^ The rapid growth of the elderly population in Japan has led to an increase in frail elderly adults. In 2001, the number of elderly adults who were approved for care was 2.6 million, which rapidly increased to 4.9 million in 2008.^1^ In this context, long-term health care facilities (hereafter referred to as nursing homes) have an important role for elderly adults and their families in Japanese society. In 2009, there were reported to be 5800 special nursing homes, with more than 400 000 residents.^2^

Activities of daily living (ADL) is one of the most important factors in characterizing the health status of frail elderly adults. Because ADL is associated with dependence, it is ordinarily evaluated to determine the levels of care that people should receive. Another aspect of ADL is its ability to predict mortality. In fact, a number of epidemiologic studies have shown that ADL levels predict mortality in elderly populations.^3^^–^^6^ In Japan, several research groups have reported that low ADL levels are associated with high mortality in elderly populations.^3^^,^^7^^–^^12^ However, these studies were conducted exclusively in community settings and not in institutional settings.

Identifying predictors of mortality among institutionalized elderly populations is important not only for residents but also for their families and caregivers who provide daily care. Such information will also help nursing home staff and physicians develop care plans and address future health care needs.^13^^,^^14^ Furthermore, it can also highlight challenges in developing a more successful care system that meets the needs of the growing frail elderly population.^5^ Accordingly, elucidation of the association between ADL level and mortality among institutionalized elderly adults will likely provide important basic information to society.

In this report, we used the Barthel Index (BI) to evaluate ADL in a large number of institutionalized elderly adults. The BI is one of the most commonly used scales worldwide due to its superior validity and reliability as a descriptive and diagnostic measure.^15^ Our aim was to determine the impact of decreased ADL on short-term mortality among a cohort of Japanese nursing home residents. Because mortality among nursing home residents is potentially much higher than that of the general population,^16^^,^^17^ we designed a 1-year, large-scale follow-up study.

## METHODS

### Participants

The participants comprised nursing home residents living in all 201 nursing homes registered with the Niigata Council of Institutions for the Elderly in Niigata Prefecture, Japan. As there are various types of nursing homes in Japan, we divided them into 2 types, based on the level of care provided: nursing homes that provide extensive care for frail elderly adults (referred to as “special nursing homes”) and nursing homes that provide minimal care for elderly adults (referred to as “other nursing homes”). In this study, we requested participation from all 201 nursing homes, which included 137 special nursing homes. We received no response from 61 nursing homes; thus a total of 140 homes participated, including 95 special nursing homes (response rate = 69.3%) and 45 other nursing homes (response rate = 70.3%). As of 1 April 2007, the cohort for this study included all 8905 elderly adults from the 140 nursing homes. Ultimately, 8902 participants were enrolled, excluding 3 for whom ADL levels could not be evaluated. The study protocol was approved by the independent Ethics Committee of Niigata University School of Medicine.

### Baseline measurements

The present study was initiated on 1 April 2007. Age, sex, height, and weight were obtained from medical records. BMI was calculated as the participant’s weight (kg) divided by the height squared (m^2^). Nurses or caregivers in charge of residents assessed ADL characteristics and dementia level at baseline, and a nurse supervising the whole assessment provided nursing home data. ADL level was determined using the BI,^18^ which consists of 10 items: feeding, transfers (bed to chair and vice versa), grooming, toilet use, bathing, mobility (on level surfaces), stair use, dressing, and bowel and bladder movement. The level of assistance (ranging from complete assistance to independence) that was required for each item was scored on a 2- to 4-point scale in which a minimal level of assistance was scored as 100 points (ie, higher score indicates lower dependence in ADL). Those with a BI score of 0 were considered totally dependent in ADL. Dementia level was assessed using the scale provided by the Ministry of Health, Labor and Welfare of Japan, which is an observer-based rating scale and is widely used in long-term care insurance systems.^19^ This scale includes 5 categories: no dementia (normal), some dementia but almost independent in daily living (I, slight), dementia with some difficulty communicating but independent in daily living with minimal observation (II, light), dementia with some difficulty communicating and requiring partial care (III, moderate), and severe dementia with difficulty communicating and requiring complete care (IV, severe).

### Follow-up

The present study was terminated on 31 March 2008. During the 1-year follow-up period, each nursing home reported information on dates of discharge and death, which was used to calculate person-years on a daily basis.

### Statistical methods

Mean values and proportions between 2 groups were tested using the *t* test and chi-square test, respectively. Mortality rate was calculated as number of deaths divided by person-years. We used the Cox proportional hazards model to estimate unadjusted and adjusted hazard ratios (HRs). Independent predictor variables in the multivariate model were selected using a stepwise method. Statistical Analysis Software (SAS; release 9.13, SAS Institute Inc., Cary, NC, USA) was used for the data analysis. A *P* value less than 0.05 was considered to indicate statistical significance.

## RESULTS

The baseline characteristics of the participants are shown in Table 1. Of the 8902 participants, 1127 died during the 1-year follow-up period. As for the frequency distribution of total BI scores, 2377 (26.7%) participants had a score of 0 (totally dependent), 1973 (22.2%) had a score of 1 to 20, 880 (9.9%) had a score of 21 to 40, 863 (9.7%) had a score of 41 to 60, 900 (10.1%) had a score of 61 to 80, 743 (8.4%) had a score of 81 to 99, and 1166 (13.1%) had a score of 100 (independent).

**Table 1.** Baseline participant characteristics

	*n*	Mean or proportion
Women (%)	6838	76.8
Age (years)	8902	84.3 (SD 8.1)
Height (cm)	8514	145.8 (SD 9.5)
Weight (kg)	8883	43.7 (SD 9.1)
Body mass index (kg/m^2^)	8510	20.6 (SD 3.8)
Total Barthel Index score	8902	38.5 (SD 38.0)
No or slight dementia (%)	2137	25.1
Normal vision (%)	7571	85.3
Normal hearing (%)	7076	79.7

Mortality rates and unadjusted HRs reported by predictor variables (ie, sex, age, weight, BMI, dementia level, type of nursing home, vision, and hearing) are shown in Table 2. All variables were significantly associated with mortality in the bivariate analysis. A multivariate proportional hazards model was also used to identify whether variables such as BI (continuous), sex (dichotomous), age (continuous), weight (continuous), BMI (continuous), level of dementia (discrete), type of nursing home (dichotomous), vision (dichotomous), and hearing (dichotomous) were independently associated with mortality. In this analysis, mortality was independently associated with BI score (adjusted HR = 0.98, 95% CI: 0.98–0.99), sex (2.0, 1.7–2.3), age (1.05, 1.04–1.06), and BMI (0.91, 0.89–0.92); however, type of nursing home, weight, dementia level, vision, and hearing were not associated with mortality.

**Table 2.** Mortality rates and unadjusted hazard ratios (HRs) according to levels of predictor variables at 1-year follow-up

Predictors	Cases	Person- years	Mortality rate (per 1000 person-years)	Unadjusted HR	95% CI
Sex					
Female (*n* = 6838)	811	6326	128	1 (reference)	
Male (*n* = 2064)	316	1861	170	1.33	1.17–1.51
				*P* < 0.001	
Age (years)					
<80 (*n* = 2309)	169	2191	77	1 (reference)	
80–89 (*n* = 4158)	512	3829	134	1.74	1.46–2.07
≥90 (*n* = 2435)	446	2167	206	2.68	2.25–3.20
				*P* for trend <0.001	
Weight					
Q1 (<38.9)	543	2464	220	3.22	2.64–3.94
Q2 (≥38.9, <44.3)	288	2107	137	1.99	1.61–2.47
Q3 (≥44.3, <51.1)	174	1914	91	1.33	1.05–1.68
Q4 (≥51.1)	116	1688	69	1 (reference)	
				*P* for trend <0.001	
Body mass index					
Q1 (<18.7)	573	2411	238	4.57	3.64–5.74
Q2 (≥18.7, <21.0)	273	1998	137	2.63	2.06–3.35
Q3 (≥21.0, <23.6)	148	1790	83	1.59	1.21–2.07
Q4 (≥23.6)	85	1626	52	1 (reference)	
				*P* for trend <0.001	
Level of dementia					
Normal or slight (*n* = 2137)	104	2042	51	1 (reference)	
Light (*n* = 1608)	153	1498	102	2.01	1.57–2.58
Moderate (*n* = 2693)	391	2455	159	3.14	2.53–3.90
Severe (*n* = 2449)	478	2177	220	4.34	3.51–5.36
				*P* for trend <0.001	
Type of nursing home					
Special (*n* = 6672)	1052	6072	173	4.91	3.88–6.19
Other (*n* = 2230)	75	2115	35	1 (reference)	
				*P* < 0.001	
Vision					
Normal (*n* = 7571)	872	6997	125	1 (reference)	
Partially or totally blind (*n* = 1306)	246	1170	210	1.70	1.47–1.95
				*P* < 0.001	
Hearing					
Normal (*n* = 7076)	780	6564	119	1 (reference)	
Partial or total hearing loss (*n* = 1802)	339	1603	211	1.79	1.57–2.03
				*P* < 0.001	

Q1, First quartile; Q2, Second quartile; Q3, Third quartile; Q4, Fourth quartile.

Adjusted HRs of mortality according to ADL level (as assessed by BI) are shown in Table 3. Except for total BI scores of 0 (totally dependent) and 100 (independent), which were observed at a relatively high frequency, total BI scores (1–99) were divided into quartiles. Total BI score was inversely related to mortality regardless of adjustment. HRs decreased as the number of adjusted covariates increased.

**Table 3.** Hazard ratios (HRs) of mortality at 1-year follow-up according to ADL levels assessed by the Barthel Index

		Quartiles					*P* for trend
		
Total Barthel Index score	0^a^	1–10 (Q1)	11–40 (Q2)	41–70 (Q3)	71–99 (Q4)	100^b^	
Number of deaths	584	214	157	102	45	25	
Person-years	2033	1326	1318	1233	1145	1133	
Mortality rate (per 1000 person-years)	287.3	161.4	119.2	82.7	39.3	22.1	
Unadjusted HR (95% CI)	13.1 (8.8–19.5)	7.4 (4.9–11.1)	5.4 (3.6–8.3)	3.8 (2.4–5.8)	1.8 (1.1–2.9)	1 (Ref.)	<0.001
Adjusted HR^c^ (95% CI)	11.5 (7.7–17.3)	6.3 (4.1–9.7)	4.5 (2.9–6.9)	3.3 (2.1–5.2)	1.6 (1.0–2.7)	1 (Ref.)	<0.001
Adjusted HR^d^ (95% CI)	8.4 (5.5–13.0)	4.9 (3.1–7.8)	3.9 (2.5–6.2)	3.1 (1.9–5.0)	1.6 (0.9–2.7)	1 (Ref.)	<0.001
Adjusted HR^e^ (95% CI)	7.6 (3.3–17.8)	3.9 (1.7–9.0)	3.5 (1.4–8.7)	2.7 (1.4–5.1)	1.3 (0.7–2.4)	1 (Ref.)	<0.001

ADL, activities of daily living.

^a^Totally dependent individuals.

^b^Totally independent individuals used as a reference group.

^c^Adjusted for sex and age.

^d^Adjusted for sex, age, and body mass index.

^e^Adjusted for sex, age, body mass index, and type of nursing home.

Adjusted HRs reported for each BI item are shown in Table 4. Level of dependency was associated with mortality for all items. The highest HR was observed for those classified as immobile in the category of “mobility”.

**Table 4.** Mortality rates and adjusted hazard ratios (HRs) for Barthel Index items at 1-year follow-up

Predictors	Cases	Person- years	Mortality rate (per 1000 person-years)	Adjusted HR^a^	95% CI
Feeding					
Independent (*n* = 5360)	380	5086	74.7	1 (reference)	
Needs help (*n* = 1078)	151	989	152.6	1.3	1.1–1.6
Unable (*n* = 2465)	596	2112	282.2	2.4	2.0–2.7
				*P* for trend <0.0001	
Transfer					
Independent (*n* = 3049)	116	2919	39.7	1 (reference)	
Major help (*n* = 1009)	106	951	111.5	1.8	1.3–2.4
Minor help (*n* = 605)	65	564	115.3	1.7	1.2–2.5
Unable (*n* = 4241)	840	3753	223.8	2.7	2.1–3.4
				*P* for trend <0.0001	
Grooming					
Independent (*n* = 2524)	94	2428	38.7	1 (reference)	
Needs to help (*n* = 6380)	1033	5760	179.4	2.1	1.6–2.7
				*P* < 0.0001	
Toilet use					
Independent (*n* = 2905)	109	2788	39.1	1 (reference)	
Needs some help (*n* = 1470)	147	1378	106.7	1.7	1.2–2.3
Dependent (*n* = 4529)	871	4021	216.6	2.7	2.1–3.6
				*P* for trend <0.0001	
Bathing					
Independent (*n* = 1671)	48	1619	29.6	1 (reference)	
Dependent (*n* = 7233)	1079	6568	164.3	2.0	1.4–2.8
				*P* < 0.0001	
Mobility					
Independent (*n* = 2145)	66	2069	31.9	1 (reference)	
Walks with help (*n* = 848)	48	796	60.3	1.6	1.0–2.3
Wheelchair independent (*n* = 1146)	96	1094	87.8	1.9	1.2–2.9
Immobile (*n* = 4765)	917	4229	216.8	3.6	2.6–5.1
				*P* for trend <0.0001	
Stairs					
Independent (*n* = 1638)	47	1584	29.7	1 (reference)	
Needs help (*n* = 1004)	53	954	55.6	1.2	0.7–2.0
Unable (*n* = 6262)	1027	5649	181.8	2.6	1.8–3.8
				*P* for trend <0.0001	
Dressing					
Independent (*n* = 2095)	62	2022	30.7	1 (reference)	
Needs help (*n* = 1953)	155	1839	84.3	1.5	1.1–2.2
Dependent (*n* = 4855)	910	4326	210.3	3.0	2.1–4.3
				*P* for trend <0.0001	
Bowels					
Continent (*n* = 2648)	100	2544	39.3	1 (reference)	
Occasional accident (*n* = 1639)	150	1540	97.4	1.5	1.1–2.0
Incontinent (*n* = 4617)	877	4102	213.8	2.4	1.8–3.2
				*P* for trend <0.0001	
Bladder					
Continent (*n* = 2517)	84	2422	34.7	1 (reference)	
Occasional accident (*n* = 1787)	168	1678	100.1	1.8	1.3–2.5
Incontinent (*n* = 4600)	875	4086	214.1	2.9	2.1–3.9
				*P* for trend <0.0001	

^a^Adjusted for sex, age, body mass index, and type of nursing home.

## DISCUSSION

Although many studies have shown ADL to be a predictor of mortality in community settings, few have included frail elderly adults.^20^ Impaired ADL has been shown to be one of the most important predictors of mortality even in frail elderly populations, but the strength of the association between the 2 variables remains poorly understood. In addition, there have been very few Asian studies on this topic. In the present study, we found a clear dose–response relationship between BI score and mortality rate after adjusting for major confounders such as sex, age, BMI, and type of nursing home. We also observed that, as compared with participants with a BI score of 100 (independent), the HR adjusted for all confounders was as high as 7.6 among those with a BI score of 0 (totally dependent), 3.9 for those with a BI score of 1 to 10, and 3.5 for those with a BI score of 11 to 40. Previous epidemiologic studies used the same instrument to assess ADL; however, their results are not directly comparable with ours due to differences in follow-up periods. For example, Kuzuya et al^21^ reported that unadjusted and adjusted HRs for a low ADL group (total BI score <55) compared with a high ADL group (total BI score ≥90) were 3.6 and 2.0, respectively, among community-dwelling frail elderly adults. Kitamura et al^12^ showed a dose–response relationship between BI score and mortality among community-dwelling elderly adults requiring care and an unadjusted relative risk of 4.8 for total BI scores less than 40 as compared with scores of 90 or higher. In other community-based studies, the relative risks of mortality for low ADL groups appeared lower.^3^^,^^13^ The association between ADL level and mortality is thus believed to be stronger among nursing home residents than among community-dwelling individuals.

Low ADL level is associated with comorbid conditions. There have been 2 reports investigating predictors of 1-year mortality in the United States. Stineman et al^17^ found that the major diseases that predicted 1-year mortality among elderly adults with varying ADL levels were stroke (HR = 1.9), coronary artery disease (1.6), chronic obstructive pulmonary disease (1.5), diabetes mellitus (1.6), and cancer (1.2). Van Dijk et al^16^ observed that renal failure (OR = 2.3), heart failure (1.5), chronic obstructive pulmonary disease (1.5), and diabetes mellitus (1.2) were significant predictors of 1-year mortality among nursing home residents. These chronic diseases are considered to be candidate covariates of ADL level. Community-based studies in Japan reported that cerebrovascular diseases increased with decreasing ADL level.^3^^,^^8^ In a stratified analysis, Fujita suggested that increased death from stroke in adults with ADL disability was due not only to aggravation or relapse of disease, but also to the occurrence of new disease.^8^ The association between ADL level and mortality in the present study may partly be explained by comorbid diseases, mainly cerebrovascular diseases.

Researchers have suggested that pneumonia is a major disease associated with decreased ADL level.^8^^,^^22^^,^^23^ In fact, infectious diseases, including pneumonia, may be an important predictor of short-term mortality. Murcia et al^23^ found that functional status predicted community-acquired pneumonia (CAP) mortality and that a total BI score of 80 or lower was associated with higher mortality among patients with CAP. Moreover, infections may be both a cause and consequence of functional impairment among nursing home residents.^22^ Therefore, infection control could be a key factor in preventing both ADL decline and mortality.

We also estimated HRs for each BI item. Among the items analyzed, mobility was most strongly associated with mortality (immobility was associated with an HR of 3.6). Therefore, mobility might be another useful parameter for predicting mortality.

Another possible factor in the association between ADL level and mortality is serum albumin level, which is considered an indicator of nutritional risk^24^ and has been shown to be an independent predictor of short-term mortality in institutionalized elderly adults.^25^ ADL and serum albumin levels were found to be closely associated with each other in their relation to mortality,^12^ although the causality of the relationships is unclear.^26^ Although we did not measure serum albumin level in the present study, it should be the focus of a future study.

Decreased cognitive function and dementia are putative predictors of mortality in elderly adults.^27^^,^^28^ We observed this correlation in bivariate but not multivariate analysis, indicating that the association was not independent. This lack of independence was due to intercorrelation between the dementia scale and total BI score (*r* = −0.68, data not shown). Previous studies also reported ADL and dementia as competing risks for mortality among frail elderly adults in institutional^16^ and non-institutional^27^ settings. Thus, dementia itself may not be an important predictor of mortality in nursing home residents.

This study has some limitations worth noting. First, disease information was not included, given the uncertainty of these data in the study population. However, such information may have allowed for more-sensitive prediction of mortality. Second, we did not evaluate interobserver reliability, although it was previously found to be adequate for the BI.^15^ This may have resulted in nondifferential misclassification of ADL levels. Third, we did not include biochemical data. For example, serum albumin and hemoglobin are promising predictors of mortality,^12^^,^^16^ and should be considered in future studies. Finally, the present study used short-term mortality as an endpoint. Thus, factors associated with long-term mortality are unknown. As was pointed out in a long-term mortality study,^8^ decreases in ADL often occur during the follow-up period, which makes it difficult to evaluate the impact of ADL on long-term mortality.

To the best of our knowledge, this is the first study to examine the association between BI score and mortality among Asian nursing home residents. We found a clear dose–response relationship between ADL level and mortality: those with low ADL levels had higher mortality. Together with other known risk factors, ADL level might effectively predict short-term mortality among elderly institutionalized adults.

## ONLINE ONLY MATERIALS

The Japanese-language abstract for articles can be accessed by clicking on the tab labeled Supplementary materials at the journal website http://dx.doi.org/10.2188/jea.JE20110153.

Abstract in Japanese.
