# Supplementary material for: Association Between Activities of Daily Living and Mortality Among Institutionalized Elderly Adults in Japan
Source: J Epidemiol. 2012 Nov 5;22(6):501–7. doi: 10.2188/jea.JE20110153 (PMC3798561; doi:10.2188/jea.JE20110153)
Supplement: Abstract in Japanese. [file je-22-501-s001.pdf]

日本の施設入居高齢者の日常生活動作（ADL）と生命予後との関連

中澤明美[1]、中村和利[2]、北村香織[2]、吉澤善明[3]
[1] 東都医療大学ヒューマンケア学部看護学科
[2] 新潟大学大学院医歯学総合研究科環境予防医学分野
[3] 新潟県老人福祉施設協議会

【背景】本研究は、日本の高齢者施設に入居している高齢者の ADL と生命予後との関連を明らかにすることを目的とした。
【方法】新潟県老人福祉施設協議会に所属する高齢者施設のうち研究の同意の得られた 140 の高齢者施設で生活する 8902 名の高齢者を対象とした 1 年間のコホート研究を実施した。ベースラインにおいて年齢、性、身長、体重、BMI、ADL と認知症レベルを調査した。ADL の程度の判定には Barthel Index(BI)を用い、施設の看護師または介護士の判定により BI の総得点（高得点ほど自立している）を算出した。退所および死亡した日付より人-年を算出した。Cox 比例ハザードモデルを用いて生命予後のハザード比とその 95%信頼区間を算出した。
【結果】対象者の平均年齢は 84.3 歳、BI 総得点の平均は 38.5 点であった。性、年齢、BMI、施設の種類で調整した生命予後のハザード比は、BI 総得点 100 点（自立）のグループを基準とすると、BI 総得点 0 点（全介助）のグループでは 7.6（95%信頼区間：3.3-17.8）、BI 総得点 1～10 点のグループでは 3.9（95%信頼区間：1.7-9.0）、BI 総得点 11～40 点のグループでは 3.5（95%信頼区間：1.4-8.7）、BI 総得点 41～70 点のグループでは 2.7（95%信頼区間：1.4-5.1）、BI 総得点 71～99 点のグループ（有意水準 p<0.001）では 1.3（95%信頼区間：0.7-2.4）であった。多変量分析では、BI、性、年齢、BMI が生命予後に関連することが明らかになった。
【結論】ADL レベルと生命予後には明確な負の関連が見られた。他のリスク要因も考慮することにより、ADL レベルは効果的に施設入居高齢者の短期間の生命予後を予測し得る。

キーワード：日常生活動作（ADL）、虚弱高齢者、高齢者施設、生命予後
